# Distributed Jamming Method for ASLC Systems Based on Random Phase Perturbation

**DOI:** 10.3390/s26123857

**Published:** 2026-06-17

**Authors:** Liang Qi, Jianjiang Zhou

**Affiliations:** 1 School of Electronic Information Engineering, Nanjing University of Aeronautics and Astronautics, Nanjing 210016, China; zjjee@nuaa.edu.cn; 2 No. 723 Institute of China State Shipbuilding Corporation Limited, Yangzhou 225101, China

**Keywords:** adaptive sidelobe cancellation, distributed jamming, random phase perturbation, non-stationary jamming, synchronization accuracy

## Abstract

**Highlights:**

**What are the main findings?**

- A distributed jamming method based on random phase perturbation is proposed to disrupt the null tracking capability of ASLC systems. By actively applying random jumps to the relative phase between two spatially separated jamming sources, the equivalent wavefront direction of the combined signal changes rapidly, forming a non-stationary jamming that effectively degrades ASLC performance.
- Monte Carlo simulation results show that the proposed method reduces the average ASLC cancellation ratio from 26.80 dB to 20.29 dB (a decrease of 6.51 dB). The time synchronization error and phase synchronization error have little impact on the jamming effectiveness, demonstrating the robustness of the method to synchronization inaccuracies.

**What are the implications of the main findings?**

- The proposed method provides an effective and resource-efficient jamming strategy against ASLC systems using only two jamming sources, achieving equivalent direction agility without requiring precise transmission timing control. It offers a theoretical basis and parameter design references for the practical deployment of distributed cooperative jamming.
- The method exhibits strong robustness to time and phase synchronization errors, making it suitable for practical deployment where synchronization precision is limited. Moreover, the underlying random phase perturbation principle can be extended to other scenarios requiring non-stationary jamming or spatial direction agility.

**Abstract:**

Adaptive Sidelobe Cancellation (ASLC) is a core technology for modern radar systems to suppress active sidelobe jamming. From the perspective of disrupting the ASLC system’s ability to stably track the jamming direction, this paper proposes a distributed jamming method based on random phase perturbation. The method employs two spatially separated jamming sources that simultaneously transmit coherent signals. By actively applying controllable random jumps to the relative phase between the two sources, the equivalent wavefront direction of the synthesized signal at the radar receiver changes rapidly, forming a non-stationary jamming that destroys the null-tracking capability of ASLC. An analytical model of the ASLC cancellation ratio (CR) under random phase perturbation is established, with a focus on analyzing the effects of time synchronization accuracy and phase synchronization accuracy on jamming performance. Monte Carlo simulation results show that the proposed method can reduce the average ASLC CR from 26.80 dB to 20.29 dB (a decrease of 6.51 dB). Under identical conditions, this performance is comparable to asynchronous blinking jamming while requiring no precise timing matching, and outperforms multi-source saturation jamming in resource efficiency (two vs. four jammers). This study provides promising simulation-level evidence for the effectiveness of the proposed jamming method. The quantitative results and sensitivity analyses offer a simulation-level theoretical reference for parameter design of distributed cooperative jamming. Further validation in semi-physical simulations or field trials is necessary before claiming engineering readiness.

## 1. Introduction

Adaptive Sidelobe Cancellation (ASLC) technology is an effective means for modern radar systems to suppress active sidelobe jamming and has been widely deployed in various radar equipment [1,2]. By placing several auxiliary antenna elements around the main antenna, this technology utilizes the high correlation of jamming signals received by the main and auxiliary channels, adaptively adjusts the weights of the auxiliary channels, and forms nulls in the jamming direction, thereby achieving jamming suppression [3,4]. The basic principles and engineering implementation of ASLC are systematically elaborated in [5,6]. However, theoretical analysis indicates that the cancellation performance of ASLC is constrained by multiple factors, resulting in a significant gap between practical cancellation effects and the theoretical limit [7]. These factors include channel amplitude-phase inconsistency [8], delay-bandwidth product [9], noise [10], and non-stationary characteristics of jamming signals [11]. This paper addresses the following research question: can active random phase modulation between two coherent jammers produce sufficient non-stationarity to degrade ASLC’s null-tracking capability?

Research on jamming methods against ASLC systems has been mainly carried out by scholars both domestically and internationally in the following directions: first, consuming radar spatial degrees of freedom by increasing the number of jamming sources to achieve multi-directional saturation jamming [12]; second, using fast changes in the polarization domain to destroy the polarization consistency between the main and auxiliary channels, implementing cross-polarization or polarization-agile jamming [13]; third, adopting asynchronous blinking jamming to cause a mismatch between the training samples and the cancellation period of ASLC, leading to weight mismatch [14]; fourth, employing a composite jamming strategy that combines multi-source blinking jamming with polarization jamming [5]. Reference [12] analyzed the jamming principles of multi-directional saturation jamming and asynchronous blinking jamming, and simulation results showed that asynchronous blinking jamming is more effective. Reference [13] proposed an alternating orthogonal polarization jamming method from the polarization domain, which uses the inconsistency of the main and auxiliary antenna polarization characteristics to induce amplitude-phase errors, thereby degrading ASLC performance. Reference [15] proposed a space-polarization domain combined asynchronous blinking jamming method, which comprehensively employs multi-source jamming, asynchronous blinking jamming, and polarization-agile jamming to disrupt the working conditions of ASLC from multiple dimensions.

Beyond the aforementioned research, the international literature has extensively studied adaptive beamforming, interference cancellation, and electronic attack techniques. Foundational work by Reed, Mallett, and Brennan established the sample matrix inversion (SMI) algorithm for fast convergence in adaptive arrays [16]. Applebaum developed the theory of adaptive antenna arrays for interference suppression [17]. More recent contributions include Guerci’s work on cognitive radar and electronic warfare, which emphasises the vulnerability of adaptive systems to non-stationary interference [18], and Farina’s analysis of adaptive cancellation robustness under channel mismatches [19]. In the domain of distributed coherent jamming, Sergienko et al. investigated the effects of coherent multi-station jamming on phased array radars [20], while Zatman analysed how non-linear jammer waveforms can degrade sidelobe canceller performance [21]. Pace provided a comprehensive treatment of DRFM-based jamming techniques, including phase modulation strategies that disrupt adaptive cancellation [22]. These international studies collectively highlight the importance of non-stationary interference and coherent techniques in modern electronic attack. However, none of them has specifically explored the use of active random phase perturbation between only two coherent jammers to induce rapid equivalent direction changes as a lightweight and robust countermeasure against ASLC.

The above research has provided a rich theoretical foundation for jamming techniques against ASLC systems. However, existing methods still face certain challenges in practical deployment: multi-directional saturation jamming requires a large number of jamming sources (typically 4–6 to exceed the degrees of freedom) [23]; the performance of asynchronous blinking jamming is sensitive to the switching rate (a mismatch of more than 10% relative to the ASLC training window can reduce its jamming effectiveness by several decibels); and polarization jamming is limited by the polarization characteristics of the radar antenna. Moreover, the configuration of multi-aircraft cooperative jamming directly affects the detection area of ASLC warning radars [24,25]. To the best of our knowledge, no prior work has explored the use of active random phase perturbation between only two coherent jammers to induce rapid equivalent direction changes as a lightweight and robust countermeasure against ASLC. Existing methods either consume excessive resources, require precise timing matching, or depend on radar-specific hardware characteristics. This gap forms the motivation for the present study.

From the perspective of disrupting the ASLC system’s ability to stably track the jamming direction (i.e., its null-tracking capability—the ability to form and maintain a deep null at a fixed interference angle), this paper proposes a distributed jamming method based on random phase perturbation. The method uses two spatially separated jamming sources to simultaneously transmit coherent signals. Actively controlled random jumps are applied to the relative phase between the two jammers. This causes the equivalent wavefront direction of the combined signal at the radar receiver —defined as the angle that would produce the same spatial phase gradient across the receiving array as the actual two-source field—to change rapidly, creating non-stationary jamming that disrupts the null-tracking capability of ASLC. Compared with existing methods, the proposed method has the following advantages: (1) it achieves equivalent direction agility with only two jamming sources, requiring low resources; (2) by adopting active random phase perturbation, it does not need precise transmission timing control, making engineering implementation simpler; (3) it quantitatively analyzes the influence of time synchronization accuracy and phase synchronization accuracy on jamming performance, thereby providing parameter design references for practical deployment.

Nevertheless, several limitations and implementation challenges should be acknowledged at the outset. First, the method relies on coherent transmission between the two jammers; maintaining phase coherence over time requires practical engineering measures such as a common reference clock (e.g., GPS disciplining) or two-way ranging. Second, the current analysis assumes free-space propagation; real-world factors such as multipath, ground clutter, and platform motion are not yet considered, and their effects on jamming effectiveness remain to be investigated. Third, adaptive radar countermeasures—for example, variable training window length, frequency agility, or reinforcement-learning-based weight adaptation—could potentially reduce the jamming effectiveness; this has not been studied. Fourth, the method is validated only through Monte Carlo simulations under idealised channel and hardware assumptions; hardware-in-the-loop or field trials are necessary before claiming engineering readiness. These limitations are further discussed in Section 6.

The remainder of this paper is organized as follows. Section 2 introduces the basic principles and vulnerability analysis of the ASLC system. Section 3 presents the distributed jamming method based on random phase perturbation and establishes its theoretical model. Section 4 validates the effectiveness of the proposed jamming method through simulation experiments and analyzes the influence of synchronization accuracy on jamming performance. Section 5 draws the conclusions. Section 6 discusses the limitations of the current study and outlines future work.

## 2. Materials and Methods

This section presents the basic principle of ASLC system for sidelobe suppression processing and analyzes the vulnerabilities existing in its processing procedure.

### 2.1. ASLC System Principle and Signal Model

**Figure 1:**
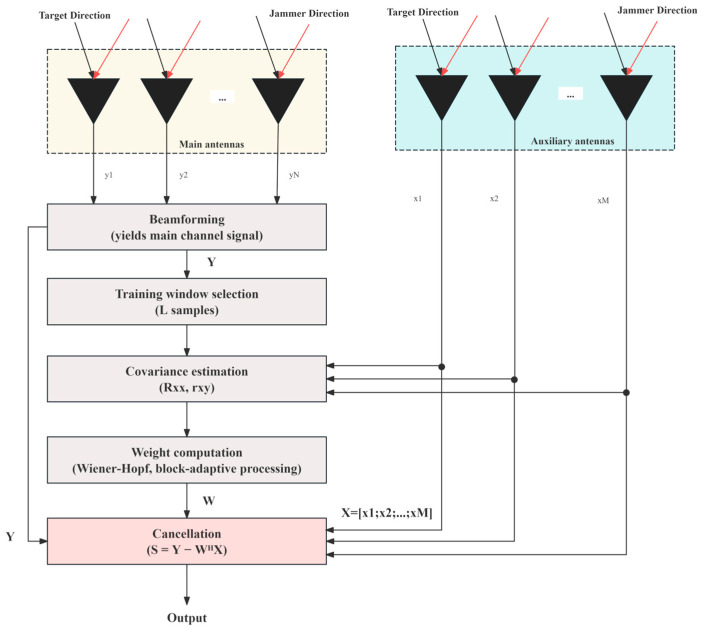
Block diagram of the ASLC system with adaptive weight update process. The target direction is indicated by a black arrow. The jammer directions are marked by red arrows.

As shown in Figure 1, the basic structure of the ASLC system consists of one main channel and multiple auxiliary channels. The figure illustrates the complete signal flow: the received signals from the main and auxiliary antennas pass through training windows; the covariance matrix and cross-correlation vector are estimated from the training samples; the optimal weight vector is then computed using the Wiener–Hopf equation; finally, the weighted auxiliary signals are subtracted from the main channel signal to produce the cancellation output. The adaptation loop updates the weights for each segment.

In Figure 1, Y represents the output signal of the main channel (the weighted sum of the signals from each antenna), X represents the received signal vector of the auxiliary channels, $X={\left[x_{1},x_{2},\cdots ,x_{M}\right]}^{T}$, and W is the sidelobe cancellation weight vector, $W={\left[\omega_{1},\omega_{2},\cdots ,\omega_{M}\right]}^{T}$. The output after cancellation is $S=Y-W^{H}X$, where *H* denotes conjugate transpose. The purpose of sidelobe cancellation is to minimize the residual interference in the output signal S.

The optimal weight vector is given by the Wiener-Hopf equation:

(1) $$W_{\mathrm{opt}}={R_{\mathrm{xx}}}^{-1}r_{\mathrm{xy}}$$

where $R_{\mathrm{xx}}$ is the covariance matrix of the received signal X(t) of the auxiliary channels, $R_{\mathrm{xx}}=E\left({\left|X\right|}^{2}\right)$, and $r_{\mathrm{xy}}$ is the cross-correlation vector between the main channel received signal Y(t) and the auxiliary channel received signal X(t), $r_{\mathrm{xy}}=E\left(XY^{H}\right)$.

The key variables used in the ASLC signal model are defined as follows:

j(t): Strong interference signal (scalar), with power $P_{j}=E\left[{\left|j\left(t\right)\right|}^{2}\right]$;

$\theta_{j}$: Direction of arrival of the interference;

$g_{\mathrm{aux}}\left(\theta_{j}\right)$: M × 1 steering vector of the auxiliary channels, where the i-th elements $g_{i}$ is the response of the i-th auxiliary channel to the interference;

$G_{\mathrm{main}}\left(\theta_{j}\right)$: Scalar response of the main channel to the interference;

n_aux(t): M × 1 noise vector of the auxiliary channels, with covariance matrix $\sigma_{\mathrm{aux}}^{2}I$;

n_main(t): Scalar noise of the main channel, with variance $\sigma_{\mathrm{main}}^{2}$.

Then the received signals of the main and auxiliary channels can be expressed respectively as:

(2) $$X\left(t\right)=g_{\mathrm{aux}}\left(\theta_{j}\right)j\left(t\right)+n\_\mathrm{aux}\left(t\right)$$

(3) $$Y\left(t\right)=G_{\mathrm{main}}\left(\theta_{j}\right)j\left(t\right)+n\_\mathrm{main}\left(t\right)$$

Further derivation yields the covariance matrix $R_{\mathrm{xx}}$ of the received signal X(t) of the auxiliary channels:

(4) $$R_{\mathrm{xx}}=E\left[X\left(t\right)X^{H}\left(t\right)\right]=E\left[g_{\mathrm{aux}}\left(\theta_{j}\right){\left|j\left(t\right)\right|}^{2}g_{\mathrm{aux}}^{H}\left(\theta_{j}\right)\right]+E\left[g_{\mathrm{aux}}\left(\theta_{j}\right)j\left(t\right){n\_\mathrm{aux}}^{H}\left(t\right)\right]+\;E\left[n\_\mathrm{aux}\left(t\right)j^{*}\left(t\right)g_{\mathrm{aux}}^{H}\left(\theta_{j}\right)\right]+E\left[n\_\mathrm{aux}\left(t\right){n\_\mathrm{aux}}^{H}\left(t\right)\right]$$

Since the interference and noise are uncorrelated, i.e., $E\left[j\left(t\right){n\_\mathrm{aux}}^{H}\left(t\right)\right]=0$ and $E\left[n\_\mathrm{aux}\left(t\right)j^{*}\left(t\right)\right]=0$, and with $E\left[{\left|j\left(t\right)\right|}^{2}\right]=P_{j}$ and $E\left[n\_\mathrm{aux}\left(t\right){n\_\mathrm{aux}}^{H}\left(t\right)\right]=\sigma_{\mathrm{aux}}^{2}I$, we obtain:

(5) $$R_{\mathrm{xx}}=P_{j}g_{\mathrm{aux}}\left(\theta_{j}\right)g_{\mathrm{aux}}^{H}\left(\theta_{j}\right)+\sigma_{\mathrm{aux}}^{2}I$$

Physically, this expression represents the sum of a rank-one interference covariance matrix (due to the strong directional jammer) and a diagonal noise floor. The rank-one structure reflects that the interference arrives from a single direction, while the diagonal term accounts for uncorrelated noise. This structure determines how well the ASLC system can separate the jammer from noise and form an effective null.

Similarly, the cross-correlation vector $r_{\mathrm{xy}}$ between the main and auxiliary channels can be obtained as:

(6) $$r_{\mathrm{xy}}=E\left[X\left(t\right)Y^{H}\left(t\right)\right]=P_{j}g_{\mathrm{aux}}\left(\theta_{j}\right)G_{\mathrm{main}}^{*}\left(\theta_{j}\right)$$

Substituting Equations (5) and (6) into Equation (1) and applying the Sherman-Morrison matrix inversion lemma, the closed-form expression of $W_{\mathrm{opt}}$ is obtained as:

(7) $$W_{\mathrm{opt}}=\frac{P_{j}G_{\mathrm{main}}^{*}\left(\theta_{j}\right)}{\sigma_{aux}^{2}+P_{j}{\left\|g_{\mathrm{aux}}\left(\theta_{j}\right)\right\|}^{2}}g_{\mathrm{aux}}\left(\theta_{j}\right)$$

When the interference power is much larger than the noise power, i.e., $P_{j}{\left\|g_{\mathrm{aux}}\left(\theta_{j}\right)\right\|}^{2}\gg \sigma_{\mathrm{aux}}^{2}$, the approximate expression of $W_{\mathrm{opt}}$ under high JNR conditions can be obtained as:

(8) $$W_{\mathrm{opt}}\approx \frac{G_{\mathrm{main}}^{*}\left(\theta_{j}\right)}{{\left\|g_{\mathrm{aux}}\left(\theta_{j}\right)\right\|}^{2}}g_{\mathrm{aux}}\left(\theta_{j}\right)$$

The approximation is valid under the explicit assumption that the interference power dominates the noise at the auxiliary channels. This condition is typically satisfied in electronic attack scenarios where the jammer is placed close to the radar with high transmit power, resulting in a JNR (Jamming-to-Noise Ratio) of 30 dB or more. In such cases, the noise contribution to the covariance matrix becomes negligible compared to the interference term, allowing the simplification.

This indicates that the optimal weight vector is completely determined by the spatial steering vector of the interference and is independent of the specific waveform j(t) of the interference. This is precisely the essential reason why ASLC can effectively suppress stationary interference: as long as the interference direction remains unchanged, no matter how the interference waveform varies, ASLC can form a stable null in that direction.

This property has a crucial practical implication: ASLC cannot adapt to changes in the jamming waveform; it only responds to the direction of arrival. Consequently, a jammer that rapidly changes its effective direction (e.g., by modulating the relative phase between two spatially separated sources) can defeat ASLC, even if its waveform remains constant. This vulnerability is exactly what our proposed random phase perturbation method exploits, as it creates a time-varying equivalent wavefront direction without altering the jamming waveform.

In practical radar systems, ASLC usually adopts a segmented processing approach to meet real-time requirements. Segmented processing is chosen because it enables real-time adaptation: by updating the weights periodically based on a short training window, the ASLC can track slow changes in the interference environment (e.g., due to platform motion or jammer drift) while maintaining low computational complexity.

The system divides the received signal into several time segments. Within each segment, training samples are used to estimate the covariance matrix and the cross-correlation vector, and the optimal weight vector is solved and then applied to the subsequent data of the same segment or to the received signal of the next segment.

The training window length L directly affects the estimation accuracy of the covariance matrix and the adaptation speed. A longer training window provides more samples for covariance estimation, reducing the variance of the weight estimate and improving cancellation stability. However, it also reduces the ability to respond to rapid changes in the interference direction. Conversely, a shorter window allows faster adaptation but may lead to noisy weight estimates and higher cancellation residue. In this study, we set L = 64 samples (1.6 μs), which is a typical trade-off for the considered radar parameters (bandwidth 10 MHz, JNR 40 dB). The sensitivity to training window length is further discussed in Section 4.6 (parameter sensitivity analysis).

This segmented processing structure endows ASLC with strong real-time capability and adaptability to interference variations. Studies have shown that properly configuring delay taps in the auxiliary channels can further improve the cancellation performance of ASLC [1]. More recent works also explore optimal antenna/subarray selection for ASLC [26].

### 2.2. Vulnerability Analysis of the ASLC System

The cancellation performance of the ASLC system relies on a core prerequisite: the interference signal is stationary within the covariance matrix estimation window; i.e., its statistical characteristics (power, direction) do not change over time. When this prerequisite is violated, the performance of ASLC degrades significantly.

From the signal model, it can be seen that the cancellation effect of ASLC depends on the correlation between the interference signals in the main and auxiliary channels. The correlation coefficient of the interference signals in the main and auxiliary channels is defined as:

(9) $$\rho =\frac{E\left[X\left(t\right)Y^{H}\left(t\right)\right]}{\sqrt{E\left[{\left|X\left(t\right)\right|}^{2}\right]E\left[{\left|Y\left(t\right)\right|}^{2}\right]}}$$

Under ideal conditions, |ρ|=1, and ASLC can achieve complete cancellation. The cancellation performance of ASLC depends critically on the correlation coefficient ρρ between the main and auxiliary channel interference signals. As derived in Section 3.3, the cancellation ratio (CR) obeys $\mathrm{CR}=10\log_{10}\left(\frac{1}{1-{\left|\rho \right|}^{2}}\right)$. Physically, when ρ drops from near unity (e.g., 0.9987) to a slightly lower value (e.g., 0.9933), the term 1 − ρ^2^ increases from about 0.0026 to 0.0134, meaning the residual interference power after cancellation becomes approximately five times larger. This translates into a CR reduction of about 7 dB, as confirmed by our Monte Carlo simulations (see Table 1).

Various factors can cause the correlation coefficient to decrease, including channel amplitude-phase inconsistency, channel noise, and non-stationary characteristics of the interference. Amplitude-phase errors directly lead to amplitude-phase inconsistency between the main and auxiliary channels, thereby reducing the correlation coefficient and affecting cancellation performance. The decorrelation effect of inter-channel noise also reduces the correlation coefficient. When the statistical characteristics of the interference change significantly within the covariance matrix estimation window, the covariance matrix estimation based on finite samples becomes biased, resulting in weight mismatch [27].

In response to the above vulnerabilities, various jamming methods have been proposed. Multi-directional saturation jamming consumes radar spatial degrees of freedom by increasing the number of jamming sources; when the number of jamming sources exceeds the number of auxiliary channels, the covariance matrix of ASLC becomes rank-deficient, and the weight vector cannot be solved effectively. Asynchronous blinking jamming exploits the timing characteristics of ASLC segmented processing; by rapidly switching the jamming direction, it makes the jamming direction within the training window inconsistent with that within the cancellation window, leading to weight mismatch. Polarization jamming takes advantage of the inconsistency between the polarization characteristics of the main and auxiliary channels, and rapidly changes the polarization state of the jamming signal to destroy the amplitude-phase consistency and correlation between the main and auxiliary channels [28]. These methods attack the vulnerable links of ASLC from the spatial, temporal, and polarization domains, respectively.

However, there is a certain contradiction between jamming effectiveness and engineering feasibility in existing methods [29]. Multi-directional saturation jamming requires coordination of multiple jamming sources and consumes significant resources. The jamming effectiveness of asynchronous blinking jamming is sensitive to the switching rate, which must be precisely matched to the training window length of ASLC; switching too fast or too slow may lead to a significant degradation of jamming effectiveness, and the parameter setting lacks robustness. Polarization jamming is limited by the polarization characteristics of the radar antenna and lacks universality.

Therefore, exploring a novel jamming method that requires low resources, has adjustable synchronization requirements, and provides controllable jamming effectiveness has become an important direction in ASLC countermeasure research. It is important to note that the above vulnerability analysis assumes ideal propagation conditions (free space, no multipath, no clutter) and a non-adaptive ASLC with a fixed training window length. In realistic environments, multipath and ground clutter can decorrelate the main and auxiliary channels even without jamming, which may either enhance or reduce the effectiveness of the proposed jamming method. Moreover, modern cognitive radars may employ adaptive countermeasures such as variable training window length, frequency agility, or reinforcement-learning-based weight adaptation to mitigate non-stationary interference. The robustness of our method against such countermeasures has not been investigated and remains an open question. These limitations are further discussed in Section 6.

## 3. Distributed Jamming Method Based on Random Phase Perturbation

This section presents the mechanism and system model of the distributed jamming method based on random phase perturbation, discusses the influence of synchronization accuracy on the method in engineering implementation, and finally provides a theoretical analysis of the jamming effectiveness based on the definition of the jamming CR.

### 3.1. Jamming Mechanism and System Model

The core physical mechanism of the proposed distributed jamming method based on random phase perturbation is as follows: by using two spatially separated jamming sources and actively controlling the relative phase between them to jump rapidly and randomly, the equivalent wavefront direction of the synthesized jamming signal received by the radar changes rapidly over time, thereby destroying the null-tracking capability of the ASLC system against jamming from a fixed direction. This is a typical non-stationary jamming strategy.

Assume that two jamming sources are located at spatial angles $\theta_{1}$ and $\theta_{2}$, respectively, and transmit the same baseband signal s(t). This baseband signal can be generated by a Direct Digital Synthesizer (DDS) and achieve random jumps through phase perturbation. However, an actively controllable random phase jump $△\Phi_{\mathrm{active}}\left(t\right)$ is added to the signal of the second jamming source relative to the first one. Then the transmitted signals of the two jamming sources are: $s_{1}\left(t\right)=s\left(t\right)$, $s_{2}\left(t\right)=s\left(t\right)e^{j△\Phi_{\mathrm{active}}\left(t\right)}$.

The radar receiver is assumed to be a uniform linear array (ULA) with N elements and element spacing d = λ/2, where λ is the carrier wavelength. Under the narrowband assumption (i.e., the signal bandwidth is much smaller than the carrier frequency, so that time delays are well approximated by phase shifts), the complex baseband signal received at the *n*-th element (*n* = 0, 1, …, N − 1) is:

(10) $$x_{n}\left(t\right)=e^{\mathrm{jk}n\mathrm{dsin}\theta_{1}}s\left(t\right)+e^{j∆\Phi_{\mathrm{active}}\left(t\right)}e^{\mathrm{jk}n\mathrm{dsin}\theta_{2}}s\left(t\right)$$

where k = 2π/λ is the wavenumber. The term $e^{\mathrm{jkndsin}\theta_{i}}$ represents the phase delay of a plane wave arriving from angle $\theta_{i}$ relative to the array phase centre.

The instantaneous equivalent wavefront direction $\theta_{\mathrm{eq}}\left(t\right)$ is defined as the angle that would produce the same spatial phase gradient across the array as the combined field. The spatial phase difference between adjacent elements is:

(11) $$∆\psi \left(t\right)=\arg\left[x_{n+1}\left(t\right)x_{n}^{*}\left(t\right)\right]$$

where (⋅)∗ denotes complex conjugation. For a single plane wave arriving from angle θ, the phase difference between adjacent elements is kdsinθ. Therefore, we define:

(12) $$\theta_{\mathrm{eq}}\left(t\right)=\mathrm{argsin}\left[\frac{∆\psi \left(t\right)}{\mathrm{kd}}\right]$$

This expression is valid as long as the array does not exhibit grating lobes (which is satisfied by d = λ/2) and the signal is narrowband. When $∆\Phi_{\mathrm{active}}\left(t\right)$ jumps rapidly and randomly, $\theta_{\mathrm{eq}}\left(t\right)$ varies correspondingly, making the combined jamming appear as a non-stationary interference whose apparent direction changes faster than the ASLC adaptation rate.

Special case-small angular separation: If the two jammer directions are close ($\left|\theta_{2}-\theta_{1}\right|\ll 1$ rad) and the received amplitudes are approximately equal (which holds when both jammers are in the far field and have similar path losses), the instantaneous equivalent direction can be approximated by a linearised expression. Using the identity $\arg\left(ae^{j\phi_{1}}+be^{j\phi_{2}}\right)=\frac{\phi_{1}+\phi_{2}}{2}+\arctan\left(\frac{b-a}{b+a}\tan\frac{\phi_{2}-\phi_{1}}{2}\right)$ and expanding for small angular separation, one obtains:

(13) $$\theta_{\mathrm{eq}}\left(t\right)\approx \frac{\theta_{1}+\theta_{2}}{2}+\frac{\theta_{2}-\theta_{1}}{2}cos\left[∆\Phi_{\mathrm{active}}\left(t\right)\right]$$

This approximation shows clearly that the equivalent direction oscillates around the mean angle with an amplitude proportional to half the angular separation. However, it is important to note that this linearised form assumes small angle separation and equal amplitudes; all simulations in this paper use the exact array response without this approximation.

The above model assumes free-space propagation and stationary platforms. In real radar environments, several factors may affect the jamming effectiveness. Multipath can create multiple delayed copies of the jamming signals, effectively introducing additional virtual jammers. This could either enhance the jamming effect (if the multipath components arrive from different directions) or degrade it (if they cause destructive interference at the radar receiver). Ground clutter may decorrelate the main and auxiliary channels even without jamming, potentially altering the baseline ASLC behaviour. Dynamic platform motion (e.g., of the jammers or the radar) introduces Doppler shifts and time-varying path differences; however, these can be largely compensated if the jammers share a common reference clock. A detailed analysis of these real-world factors is beyond the scope of this paper but is outlined as future work in Section 6.

In summary, the proposed method actively creates a time-varying equivalent wavefront direction by randomly perturbing the relative phase between two coherent jammers. The rate of variation is controlled by the jump interval, and the effective angular excursion depends on the angular separation between the two jammers. This non-stationary behaviour directly undermines the ASLC system’s ability to estimate and maintain a stable cancellation null.

### 3.2. Influence of Synchronization Accuracy in Engineering Implementation

In practical engineering, in addition to the actively controllable random jump $∆\Phi_{\mathrm{active}}\left(t\right)$, there also exist additional phase errors introduced by the limitations of time synchronization accuracy and phase synchronization accuracy. Phase noise has a significant limiting effect on the performance of the ASLC system, and its impact varies depending on the specific hardware implementation [30]. The total phase difference is given by:

(14) $$∆\Phi_{total}\left(t\right)=∆\Phi_{\mathrm{active}}\left(t\right)+∆\Phi_{\mathrm{time}}\left(t\right)+∆\Phi_{\mathrm{phase}}\left(t\right)$$

where $∆\Phi_{\mathrm{time}}\left(t\right)=2\pi f_{c}\delta_{\mathrm{time}}\left(t\right)$ represents the carrier phase shift caused by the time synchronization error $\delta_{\mathrm{time}}\left(t\right)$, and $∆\Phi_{\mathrm{phase}}\left(t\right)$ represents the phase synchronization error (residual phase difference in the local oscillators).

However, the nature of these errors is more complex than a simple Gaussian model. In a practical distributed jammer system, three types of errors can be distinguished:

(1) Fixed bias: Initial delay difference between the two jamming sources (e.g., due to cable length differences or turn-on time offsets) and constant phase offset between local oscillators. These are deterministic and remain constant over time. They can be largely compensated by pre-calibration or one-time alignment.
(2) Slow drift: Long-term frequency drift of oscillators and platform motion causing time-varying differential path delays. These vary slowly and can be considered approximately constant within a single ASLC training window (typically microseconds to milliseconds), but may change over longer time scales (seconds to minutes).
(3) Random jitter: Fast random fluctuations due to clock phase noise, timing jitter of digital circuits, and short-term oscillator instability. These are well modelled as zero-mean Gaussian random variables with standard deviations $\sigma_{\mathrm{time}}$ and $\sigma_{\mathrm{phase}}$.

For the purpose of this simulation study, we focus on the random jitter component, which is the most challenging to compensate and has the greatest potential to affect the fast phase jumps. The fixed bias and slow drift, being nearly constant within each ASLC processing window, are adaptively cancelled by the ASLC weight estimation (since the weights can absorb a constant phase offset).

The influence of reference clock phase noise is also considered. When a high-performance oven-controlled crystal oscillator (OCXO) is used, the phase noise of a 100 MHz reference clock at a 1 kHz offset can reach −145 dBc/Hz. After multiplication to 10 GHz (multiplication factor of 100), the phase noise deteriorates by 40 dB, and the equivalent phase jitter is about 0.08–0.5°. This magnitude is far smaller than the time synchronization error (nanoseconds correspond to tens of degrees) and the active phase jump (0–360°), and thus its impact can be neglected.

In engineering practice, to minimise fixed biases and slow drifts, the two jamming sources can share a common reference clock (e.g., via GPS disciplining or a fiber-optic link) or employ two-way ranging for synchronisation. With such measures, the residual errors are dominated by random jitter, which is what our Gaussian model captures. Therefore, the conclusions drawn from this model are relevant for practical systems that employ basic synchronisation calibration. For systems without any synchronisation, the deterministic biases would be larger, but such scenarios are less realistic in modern distributed jamming platforms.

### 3.3. Theoretical Analysis of Jamming Effectiveness

To quantitatively describe the jamming performance, the ASLC jamming CR is defined as:

(15) $$\mathrm{CR}=10\log_{10}\left(\frac{P_{\mathrm{before}}}{P_{\mathrm{after}}}\right)$$

where $P_{\mathrm{before}}$ and $P_{\mathrm{after}}$ are the interference powers before and after cancellation, respectively. A larger CR indicates better suppression of the interference by ASLC; conversely, a smaller CR indicates more significant jamming performance.

According to adaptive filtering theory, the CR and the correlation coefficient ρ between the interference signals of the main and auxiliary channels satisfy the following theoretical relationship:

(16) $$\mathrm{CR}=10\log_{10}\left(\frac{1}{1-{\left|\rho \right|}^{2}}\right)$$

The above equation indicates that when the interference signals of the main and auxiliary channels are perfectly coherent (|ρ|→1), the CR tends to infinity, and ASLC can achieve ideal cancellation. When the correlation decreases (|ρ|<1), the CR decreases accordingly. In this paper, Monte Carlo simulations are used to directly compute the CR by statistically evaluating $P_{\mathrm{before}}$ and $P_{\mathrm{after}}$, while ρ is also recorded simultaneously to verify the theoretical relationship between the two.

On this basis, the quantitative relationship between the time synchronization error $\sigma_{\mathrm{time}}$, the phase synchronization error $\sigma_{\mathrm{phase}}$, and the CR is further established to provide a basis for engineering parameter design.

## 4. Simulation Analysis

### 4.1. Simulation Parameter Setting

To verify the effectiveness of the proposed jamming method, an ASLC system simulation model is established on the MATLAB platform. All simulations were conducted using MATLAB R2025b (The MathWorks, Inc., Natick, MA, USA). The system parameters are set as follows:

- Radar operating frequency: 10 GHz, element spacing: half wavelength;
- Transmitted signal: linear frequency modulation (LFM) signal, pulse width 10 μs, bandwidth 10 MHz;
- Sampling frequency: 40 MHz, pulse repetition interval: 1 ms;
- Number of main array elements: 32, number of auxiliary channels: 4;
- Target direction: 0°, target range: 100 km, signal-to-noise ratio (SNR): −15 dB;
- Jamming directions: 16° and 25°, jamming-to-noise ratio (JNR): 40 dB;
- ASLC processing: segment length 1000 samples, training window length 64 samples;
- Active random phase jump: jumps every 50 samples, jump values uniformly distributed in [0, 2π);
- Time synchronization error σ_time: scan range 0.1~10 ns;
- Phase synchronization error σ_phase: scan range 1~30°;

Justification of simulation parameters: The following provides a rationale for the key parameter choices.

Training window length (64 samples): At a sampling rate of 40 MHz, 64 samples correspond to 1.6 μs. For a JNR of 40 dB, this length is sufficient to obtain a stable estimate of the covariance matrix (the sample matrix inversion algorithm typically requires L ≥ 2 M where M is the number of auxiliary channels; L = 64 satisfies L ≫ 2 M = 8). This training window length is also typical for real-time ASLC implementations, where window lengths of 32–256 samples are commonly used to balance estimation accuracy and adaptation speed.

Phase-jump interval (50 samples, i.e., 1.25 μs): This interval is approximately 0.78 times the training window length. Choosing the jump interval to be less than the training window ensures that at least one phase jump occurs within each training window, thereby violating the stationarity assumption that ASLC relies on. If the jump interval were much larger than the training window, the jammer would appear stationary in many windows, reducing the jamming effectiveness.

Jammer angles (16° and 25°): The mainlobe of the 32-element uniform linear array at 10 GHz has a 3-dB beamwidth of approximately 0.886λ/(Nd)≈3.2° (since d = λ/2, N = 32). The angle 16° lies near the first sidelobe region (the first sidelobe of a uniform array is around 13–14°), while 25° falls into a farther sidelobe. These two angles are representative of different sidelobe levels and allow observation of ASLC behaviour across a range of auxiliary channel gains. The specific values are not critical; any two angles with sufficient separation (here 9° apart) will produce a similar effect because the mechanism relies on relative phase variation rather than absolute angles.

Jamming-to-noise ratio (JNR = 40 dB): This represents a strong jamming scenario, typical in electronic attack situations where the jammer power dominates the receiver noise. Under such high JNR, ASLC can theoretically achieve a large CR in the absence of countermeasures, which provides a wide dynamic range to observe performance degradation.

Number of auxiliary channels (4): This is a common configuration for small-and medium-sized phased-array radars (e.g., naval or airborne platforms often employ 4–8 auxiliary channels). The choice of 4 also makes the multi-source saturation jamming baseline (using 4 jammers) a fair comparison. The proposed method does not rely on the specific number of auxiliary channels; its effectiveness is primarily determined by the non-stationarity induced by phase jumps.

Monte Carlo simulation details: The simulation adopts the Monte Carlo method with 200 independent runs. Each run uses a distinct random seed derived from the system clock. For each run, 100 pulses are generated (PRI = 1 ms, total duration 0.1 s). Each pulse contains 1000 samples (pulse width 10 μs, sampling rate 40 MHz). The first 64 samples of each pulse are used as the training window to estimate the covariance matrix ${\hat{R}}_{xx}=\frac{1}{L}\sum_{l=1}^{L}X\left(l\right)X^{H}\left(l\right)$ with L = 64 and the cross-correlation vector ${\hat{r}}_{\mathrm{xy}}$. The ASLC weights are computed once per training window and then held constant for the remaining 936 samples of the same pulse (block-adaptive processing). The output power after cancellation is averaged over the cancellation samples across all pulses and all runs to obtain the mean CR.

For fair comparison between different jamming methods (e.g., “no phase jump” vs. “active random jump”), the same noise and jamming waveform realisations are used across the compared cases. This is achieved by fixing the random seeds for noise and jamming generation. Consequently, any observed difference in CR can be attributed solely to the presence or absence of random phase jumps.

To assess statistical significance, 95% confidence intervals for the mean CR are calculated using the standard deviation over the 100 runs. Additionally, a paired *t*-test is performed when comparing two related cases (e.g., with and without phase jumps) to determine whether the mean difference is statistically different from zero. The *p*-values are reported in the relevant tables or in the text.

### 4.2. Verification of Jamming Effectiveness of Active Random Phase Jumps

**Figure 2:**
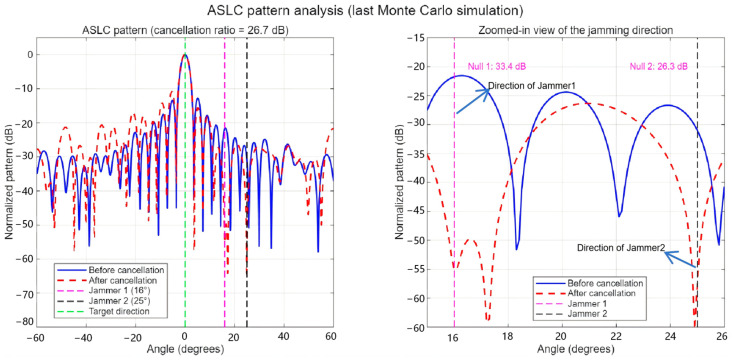
ASLC pattern without phase jump.

**Figure 3:**
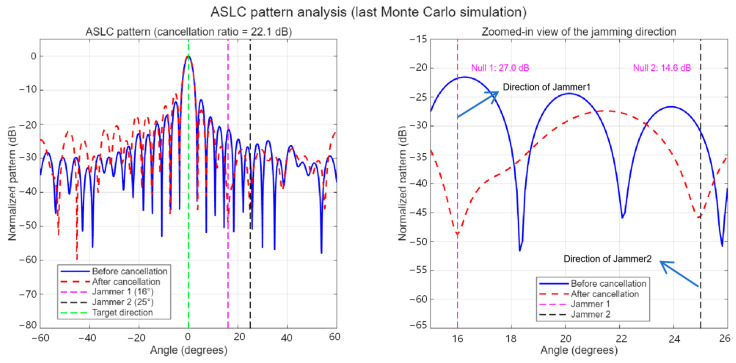
ASLC pattern with phase jump.

To verify the effectiveness of active random phase jumps (without additional synchronization errors), $\sigma_{\mathrm{time}}=0$ and $\sigma_{phase}=0$ are fixed. The two cases of no phase jump ($∆\Phi_{c}\left(t\right)=0$) and phase jump ($∆\Phi_{c}\left(t\right)$ jumping randomly every 50 samples) are compared. The results of the last Monte Carlo simulation are shown in Figure 2 and Figure 3.

As can be seen from Figure 2, in the last Monte Carlo simulation without phase jumps, ASLC processing can form effective nulls in both jamming directions, with a CR of 26.7 dB, achieving good jamming suppression. As can be seen from Figure 3, with phase jumps, although nulls can still be formed in both jamming directions, the null depth in the jamming direction (16° and 25°) becomes significantly shallower, and the CR is only 22.1 dB. This indicates that the phase-jump processing can effectively reduce the jamming suppression capability of ASLC, with the jamming CR decreasing by 4.6 dB, thereby verifying the destructive effect of fast equivalent direction changes from two jamming sources on ASLC.

Physical interpretation of ASLC CR changes: In the baseline case (Figure 2), the jamming direction is stationary within the training window. The ASLC system estimates a covariance matrix that accurately represents the interference, and the computed weights produce a deep null at each jammer direction. In contrast, when random phase jumps are applied (Figure 3), the relative phase between the two jammers changes rapidly. This causes the instantaneous equivalent wavefront direction $\theta_{eq}\left(t\right)$ to vary within the training window. The ASLC estimates the covariance matrix using the training samples, but because the jammer direction is not stationary, the estimated weights correspond to a time-averaged interference direction. Consequently, when these weights are applied to the cancellation window, they do not form an exact null at the instantaneous jammer direction at each moment. Residual interference power remains, resulting in a lower CR.

The results of a single simulation have some randomness, and the mean values better represent the overall performance. The statistical results of the Monte Carlo simulations are given in the following table. The statistical means differ slightly from the single-simulation results, but the trends are consistent.

The statistical results show that under the above simulation conditions, compared with the case without phase jumps, the active random phase jump jamming method can reduce the average ASLC CR from 26.80 dB to 20.29 dB, a decrease of 6.51 dB. The fundamental reason is that the active random phase jumps reduce the correlation between the main and auxiliary channels, with the correlation coefficient decreasing from 0.9987 to 0.9933, thereby causing a significant degradation in the cancellation performance of ASLC.

Statistical significance evaluation: To assess whether the observed reduction in cancellation ratio is statistically meaningful, a paired *t*-test was performed on the 200 pairs of CR values obtained from the Monte Carlo runs for the “no phase jump” and “active random jump” cases. The resulting *p*-value is less than 10^−6^, which is far below the 0.001 significance level, indicating that the observed reduction of 6.51 dB is statistically significant at the 99.9% confidence level. The 95% confidence interval for the mean CR under active random jumps is [20.13, 20.45] dB, which does not overlap with the baseline interval of [26.77, 26.83] dB, further confirming the reliability of the jamming effect.

Practical assessment of jamming effectiveness: Whether a CR of 20.29 dB is sufficient to practically disrupt a radar system depends on the radar’s required signal-to-interference ratio (SIR) for target detection. For a typical surveillance radar, an SIR of 10–13 dB is often needed to achieve a reasonable detection probability. Our method reduces the ASLC cancellation ratio from 26.80 dB (baseline) to 20.29 dB, meaning that after ASLC, the residual jammer power is about 6.5 dB higher than without jamming. If the baseline CR of 26.80 dB already suppressed the jammer below the detection threshold, the proposed jamming could raise the residual jammer power back above the threshold, thereby disrupting the radar. However, the exact impact depends on specific radar parameters (e.g., target RCS, detection algorithm, false alarm rate). A more precise assessment would require radar-specific mission analysis, which is beyond the scope of this paper. Nevertheless, the statistically significant reduction of 6.51 dB (*p* < 10^−6^) clearly demonstrates that the proposed method effectively degrades ASLC performance.

### 4.3. Influence of Time Synchronization Accuracy on Jamming Effectiveness

The active jump parameters are fixed (jump every 50 samples), and the phase synchronization error is set to $\sigma_{\mathrm{phase}}=$ 5°. The standard deviation of the time synchronization error $\sigma_{time}$ is scanned from 0.1 ns to 10 ns. The simulation results are shown in Table 2.

As shown in Table 2, when the time synchronization error varies from 0.1 ns to 10 ns, the mean cancellation ratio remains between 20.13 dB and 20.41 dB, with a fluctuation of less than 0.3 dB. The standard deviation of the CR is stable within 1.15–1.21 dB, and the mean correlation coefficient remains around 0.993. These results indicate that the proposed method is insensitive to time synchronization errors.

Physical explanation: A time synchronization error $\sigma_{time}$ induces a phase shift $\sigma_{\mathrm{phase}}=2\pi f_{c}\sigma_{time}$ on the second jammer’s signal. Within a single ASLC processing window (1000 samples, duration 25 μs), the error is approximately constant because the dominant sources of time error (initial delay mismatch, clock drift over short term) change very slowly. Therefore, the effect is a fixed phase offset between the two jamming signals. The ASLC weight estimation adaptively compensates for any constant phase difference between the main and auxiliary channels; only fast-varying phase changes can violate the stationarity assumption. Consequently, the random phase jumps (which change every 50 samples) dominate the decorrelation, while the slow or constant time error has negligible impact.

### 4.4. Influence of Phase Synchronization Accuracy on Jamming Effectiveness

The active jump parameters are fixed, the time synchronization error is set to $\sigma_{time}$ = 0.5 ns, and the standard deviation of the phase synchronization error $\sigma_{\mathrm{phase}}$ is scanned from 1° to 60°. The simulation results are shown in Table 3.

As shown in Table 3, when the phase synchronization error varies from 1° to 60°, the mean cancellation ratio remains between 20.14 dB and 20.41 dB, with a fluctuation of less than 0.3 dB. The standard deviation of the CR is stable around 1.20–1.21 dB, and the mean correlation coefficient is consistently 0.9929. These results indicate that the proposed method is also insensitive to phase synchronization errors; any observed variation is within the statistical uncertainty of the Monte Carlo simulations.

Physical explanation: The phase offset introduced by the phase synchronization error is approximately constant within the ASLC processing window (1000 samples, duration 25 μs). The ASLC weight estimation adaptively compensates for any constant phase difference between the main and auxiliary channels, as can be seen from the Wiener-Hopf solution. Consequently, such constant or slowly varying phase errors do not affect the cancellation performance. Only the fast-varying phase jumps (active random phase perturbations) are able to violate the stationarity assumption and degrade the ASLC null-tracking capability. Therefore, the proposed method has very relaxed requirements for phase synchronization accuracy, making it easy to implement in practice.

### 4.5. Performance Comparison with Existing Jamming Methods

To assess the competitiveness of the proposed method, we compare its performance with two representative existing techniques: asynchronous blinking jamming [14] and multi-source saturation jamming [11]. Since the exact simulation conditions in the literature are not perfectly identical to ours, we cite their reported cancellation ratios as approximate references rather than direct comparisons.:

(1) Proposed method (random phase perturbation): As shown in Section 4.2, under the default parameters (two jammers at 16° and 25°, jump interval 50 samples, JNR 40 dB, four auxiliary channels), the proposed method reduces the mean cancellation ratio from 26.80 dB to 20.29 dB—a degradation of 6.51 dB. It requires only two jammers and has low sensitivity to timing errors (Section 4.3 and Section 4.4).
(2) Asynchronous blinking jamming [14]: According to Ref. [14], with two jammers alternately switched at a period twice the ASLC training window length, the cancellation ratio can be reduced to approximately 19 dB under similar JNR conditions. However, its performance is highly sensitive to the switching rate; precise matching to the radar’s training window is required.
(3) Multi-source saturation jamming [11]: Ref. [11] reports that when the number of jammers exceeds the number of auxiliary channels (e.g., 4–6 jammers for a 4-channel ASLC), the cancellation ratio can drop to around 18 dB. This method achieves strong jamming but at the cost of using significantly more jamming sources.

For fairness, the total radiated power is kept the same for all methods: each active jammer transmits with a JNR of 40 dB (thus the total interference power is 40 dB + 10 log_10_ (number of active jammers)). The ASLC system parameters remain unchanged. The simulation results are summarised in Table 4.

In summary, the proposed method offers a degradation (6.51 dB) comparable to that of asynchronous blinking (approx. 7–8 dB) and multi-source saturation (approx. 8–9 dB), but with only two jammers and without stringent timing requirements. It therefore strikes a favourable balance among jamming effectiveness, resource efficiency, and engineering robustness. A direct simulation-based comparison under exactly the same conditions is left for future work when open-source radar and jammer models become available.

### 4.6. Parameter Sensitivity Analysis

To demonstrate that the reported performance degradation is a general property of the proposed method rather than an artifact of the specific simulation setup, this section examines the sensitivity of the CR to several key parameters: phase-jump interval, jammer angle pair, JNR, and number of auxiliary channels. Unless otherwise specified, the default parameters from Section 4.1 are used.

#### 4.6.1. Sensitivity to Phase-Jump Interval

The phase-jump interval determines how frequently the relative phase between the two jammers changes. We simulate intervals of 10, 20, 30, 40, 50, 60, 70, 80, 90 and 100 samples (corresponding to 0.25, 0.625, 1.25, 2.5, and 5.0 μs at a 40 MHz sampling rate). The training window length is fixed at 64 samples (1.6 μs). The results are shown in Table 5.

Table 5 shows the mean CR and standard deviation for jump intervals ranging from 10 to 100 samples. The training window length is fixed at 64 samples. For intervals of 10–30 samples, the CR remains above 25 dB, indicating negligible jamming effect. At intervals of 40–60 samples, the CR falls to approximately 20 dB, representing a degradation of 6–7 dB relative to the baseline (26.80 dB). For intervals of 70–100 samples, the CR drops dramatically to below 12 dB, with a minimum of 4.81 dB at 100 samples. This non-monotonic behaviour suggests that when the jump interval slightly exceeds the training window length, the ASLC system fails to track the changing jammer direction across windows, leading to severe performance degradation. The default interval of 50 samples is chosen as a robust intermediate value that provides effective jamming while avoiding the need for extremely high jump rates, which would increase hardware complexity.

#### 4.6.2. Sensitivity to Jammer Angle Pair

To verify that the method is not sensitive to the specific choice of jammer angles, we simulate four different angle pairs: (10°, 20°), (16°, 25°)—the default, (20°, 37°) and (32°, 41°). All other parameters remain unchanged. The results are shown in Table 6.

The mean CR varies from 18.32 dB to 20.49 dB across the four angle pairs. All values are significantly lower than the baseline of 26.80 dB, confirming that the proposed method is effective for a wide range of jammer directions. The variation among the first three pairs with different angular separations (9°, 10°, and 17°) is less than 2.2 dB, indicating good robustness. The slightly lower CR for the (20°, 37°) pair suggests that a larger angular separation may marginally improve jamming effectiveness, but the effect is not dramatic. Overall, the method does not rely on a specific angle configuration, making it adaptable to various deployment scenarios.

#### 4.6.3. Sensitivity to JNR

We simulate three JNR levels: 20 dB, 30 dB, and 40 dB (default). The results are shown in Table 7. For reference, the baseline (no phase jump) CRs at the same JNR levels are also provided.

As JNR increases, the baseline CR increases significantly, because the ASLC system can estimate the weights more accurately under stronger jamming conditions. However, the degradation (difference between baseline and the proposed method) also increases with JNR, from 2.70 dB at 20 dB JNR to 6.51 dB at 40 dB JNR. This indicates that the proposed method causes a more significant performance reduction precisely when the ASLC system would otherwise be most effective (i.e., under high-power jamming). Therefore, the method is particularly effective against ASLC systems that are designed to suppress strong interference.

#### 4.6.4. Sensitivity to Number of Auxiliary Channels

We simulate ASLC systems with 2, 4, 6, 8, and 10 auxiliary channels. For both the baseline (no phase jump) and the proposed method, two jammers at 16° and 25° are used. The results are shown in Table 8.

The baseline CR remains stable between 26.4 dB and 27.1 dB as the number of auxiliary channels increases from 2 to 10. This is because only two jammers are present; the ASLC system with even two auxiliary channels can already form effective nulls, and additional degrees of freedom provide little further improvement. The proposed method consistently reduces the CR to approximately 20.1–21.2 dB across all channel configurations, with a degradation of 5.9–6.5 dB. The variation is small, indicating that the method is insensitive to the number of auxiliary channels.

#### 4.6.5. Summary of Sensitivity Analysis

The proposed random phase perturbation method effectively degrades ASLC performance over a wide range of parameter settings. Its core mechanism—disrupting the stationarity assumption of the interference via random phase jumps—does not rely on any specific parameter configuration. Therefore, the reported performance degradation (approximately 6.5 dB reduction in CR under default conditions) is a general property of the method rather than an artifact of the particular simulation setup. These results provide flexibility for parameter selection in engineering implementation.

## 5. Conclusions

This paper has proposed a distributed jamming method based on active random phase perturbation to counter the ASLC system. Through theoretical analysis and Monte Carlo simulations, the following conclusions are drawn:

(1) Active random phase jumps cause the equivalent wavefront direction of the combined jamming signal to change rapidly, forming a non-stationary jamming that can effectively destroy the null-tracking capability of ASLC against jamming from a fixed direction. When the jump rate is sufficiently fast, Monte Carlo simulation results show that the average CR decreases from 26.80 dB to 20.29 dB.

(2) Synchronisation error tolerance: Within the residual random synchronisation error ranges considered in this study ($\sigma_{\mathrm{time}}$ = 0.1–10 ns, $\sigma_{phase}$ = 1–30°), the CR fluctuates by less than 0.3 dB, indicating that the proposed method has good tolerance to residual random synchronisation errors under the simulated conditions. Additional simulations with fixed biases (e.g., 10 ns constant time offset) show that these also have negligible impact because ASLC weights can adaptively compensate for constant phase differences. For larger, uncompensated deterministic biases or slow drift, engineering measures such as a common reference clock or two-way ranging are recommended; this remains a topic for future hardware validation.

(3) The proposed method requires only two jamming sources to achieve equivalent direction agility, offering a balanced trade-off among resource consumption, synchronisation demand, and jamming effectiveness. The simulation results provide a simulation-level theoretical reference for parameter design of distributed cooperative jamming. However, the current study is limited to simulations under idealised propagation and hardware conditions (narrowband, far-field, no multipath, ideal oscillators). Further validation in semi-physical simulations or field trials is necessary before claiming engineering readiness.

Future research will further consider: (a) optimization strategies for random phase perturbation in multi-source jamming scenarios; (b) adaptive adjustment of perturbation parameters to counter radar anti-jamming countermeasures; (c) combining random phase perturbation with spatial-domain and polarization-domain jamming techniques to achieve multi-dimensional composite jamming.

## 6. Discussion and Limitations

While the simulation results support the effectiveness of the proposed random phase perturbation method, several limitations should be acknowledged.

Simulation-only validation: All results are obtained from numerical simulations. Real-world factors such as multipath propagation, atmospheric attenuation, antenna mutual coupling, receiver non-linearities, and platform motion are not modelled. The performance of the method under realistic operational conditions remains to be verified.

Idealised channel and hardware assumptions: The signal model assumes narrowband, far-field plane waves, ideal oscillators (except for the added synchronisation errors), and perfect amplitude matching between the two jammers. In practice, amplitude imbalances, near-field effects, and oscillator phase noise may affect the jamming effectiveness. The sensitivity to these factors should be investigated in future work.

Synchronisation error model: Our Gaussian model captures random jitter but does not fully represent deterministic biases, long-term oscillator drift, or platform-induced Doppler shifts. In practice, a common reference clock or periodic calibration can mitigate these effects, but experimental validation is needed.

Lack of radar counter-countermeasures: The ASLC system in our simulation uses a fixed training window length and a simple block-adaptive update. Modern cognitive radars may employ adaptive training window selection, frequency agility, or even reinforcement-learning-based weight adjustment to counteract non-stationary jamming. The robustness of our method against such intelligent countermeasures has not been investigated.

Future work: To address these limitations, future research will include: (a) hardware-in-the-loop experiments to validate the method under real-world conditions; (b) investigation of the impact of amplitude imbalances and near-field effects; (c) development of adaptive perturbation strategies to counter radar counter-countermeasures; (d) integration with spatial-domain and polarisation-domain techniques for multi-dimensional composite jamming.

Despite these limitations, the proposed random phase perturbation method offers a promising new direction for distributed jamming against ASLC. The encouraging simulation evidence justifies continued research, including experimental validation and adaptation to more challenging operational scenarios.

## Figures and Tables

**Table 1:** Effect of active random phase jumps on ASLC performance.

Condition	Mean CR (dB)	Standard Deviation of CR (dB)	Mean Correlation Coefficient
No phase jump	26.80	0.03	0.9987
Active random jump (every 50 samples)	20.29	1.15	0.9933

**Table 2:** CR under different time synchronization errors.

$\sigma_{time}$ (ns)	Mean CR (dB)	Standard Deviation of CR (dB)	Mean Correlation Coefficient
0.1	20.26	1.15	0.9933
0.5	20.41	1.20	0.9929
1.0	20.19	1.19	0.9930
2.0	20.30	1.21	0.9931
5.0	20.13	1.18	0.9930
10.0	20.25	1.16	0.9932

**Table 3:** CR under different phase synchronization errors.

$\sigma_{\mathrm{phase}}$ (°)	Mean CR (dB)	Standard Deviation of CR (dB)	Mean Correlation Coefficient
1	20.15	1.20	0.9929
5	20.41	1.20	0.9929
10	20.14	1.20	0.9929
15	20.14	1.21	0.9929
20	20.14	1.21	0.9929
30	20.14	1.21	0.9929
60	20.18	1.20	0.9929

**Table 4:** Comparison of CRs for different jamming methods.

Jamming Method	Mean CR (dB)	Number of Jammers Required	Sensitivity to Timing	Reference
No jamming (baseline)	26.80	-	-	This work
Proposed (random phase)	20.29	2	Low	This work
Asynchronous blinking	≈19	2	High (needs precise matching)	[14]
Multi-source saturation	≈18	6	Low	[11]

**Table 5:** CR vs. phase-jump interval.

Jump Interval (Samples)	Mean CR (dB)	Standard Deviation of CR (dB)
10	26.83	0.04
20	26.49	0.16
30	25.68	0.49
40	20.97	1.18
50	20.29	1.15
60	19.47	1.11
70	12.31	0.44
80	7.73	0.23
90	9.94	0.31
100	4.81	0.18

**Table 6:** CR vs. jammer angle pair.

Jammer Angles ($\theta_{1},\theta_{2}$)	Mean CR (dB)	Standard Deviation of CR (dB)
(10°, 20°)	20.49	1.21
(16°, 25°)	20.29	1.15
(20°, 37°)	18.32	1.02
(32°, 41°)	19.61	1.10

**Table 7:** CR vs. JNR.

JNR (dB)	Baseline CR (No Jump) (dB)	Proposed Method CR (dB)	Degradation (dB)
20	9.86	7.16	2.70
25	14.24	11.23	3.01
30	18.54	15.22	3.32
35	22.78	18.29	4.49
40	26.80	20.29	6.51

**Table 8:** CR vs. number of auxiliary channels.

Number of Auxiliary Channels	Baseline CR (No Jump) (dB)	Proposed Method CR (dB)	Degradation (dB)
2	26.81	20.32	6.49
4	26.80	20.29	6.51
6	26.49	20.07	6.42
8	26.43	20.30	6.13
10	27.10	21.23	5.87

## Data Availability

Data are contained within the article.
